# The Association Between Cannabis Use and Schizophrenia: Causative or Curative? A Systematic Review

**DOI:** 10.7759/cureus.9309

**Published:** 2020-07-21

**Authors:** Shweta Patel, Sahar Khan, Saipavankumar M, Pousettef Hamid

**Affiliations:** 1 Psychiatry, California Institute of Behavioral Neurosciences & Psychology, Fairfield, USA; 2 Internal Medicine, California Institute of Behavioral Neurosciences & Psychology, Fairfield, USA; 3 Pediatrics, California Institute of Behavioral Neurosciences & Psychology, Fairfield, USA; 4 Neurology, California Institute of Behavioral Neurosciences & Psychology, Fairfield, USA

**Keywords:** marijuana, cannabis, schizophrenia, psychosis, mental health, schizophrenia spectrum, thc, cbd

## Abstract

Marijuana is one of the most abused substances in the world. Marijuana is getting legalized around the world. So, it is crucial to understand its effect on our mental health. Its impact on the schizophrenia spectrum needs our special attention. Even though marijuana has been around for a long time, its exact effects are still unknown. Schizophrenia is a chronic illness affecting approximately 20 million people worldwide. Schizophrenia and cannabis seem to have a close relationship, and we want to explore this. We want to know if marijuana is causing, exacerbating, or treating schizophrenia. This systematic review explores this question. We searched online resources like PubMed, PubMed Central, Cochrane Library, and Google Scholar for systematic reviews, traditional reviews, randomized controlled trials, and meta-analysis on cannabis and schizophrenia/ psychosis. We included human studies published in peer-reviewed journals in the English language in the last five years. After reviewing 96 initial results of our search, we excluded 25 duplicates, 29 abstracts, and 18 irrelevant articles. We did a quality assessment for the remaining 24 studies using various quality assessment tools. After the quality assessment, we found 12 articles were of low quality and excluded those. We included the remaining 12 final studies in our systematic review. Out of these 12 studies, five were traditional reviews, two systematic reviews, two meta-analysis, and three observational studies. Six of the articles were on cannabis’s effect on just schizophrenia or psychotic disorder. The other six included schizophrenia plus other psychiatric or neurological illnesses. Ten of the studies had data supporting the causative link between cannabis and schizophrenia. Eight records had data supporting the exacerbating effect of marijuana. Six studies had data supporting the therapeutic effect of the cannabidiol (CBD) component of cannabis. From the current data, we can conclude that the tetrahydrocannabinol (THC) component of cannabis can be the main culprit causing psychosis and schizophrenia in the at-risk population. THC can also be the one exacerbating symptoms and causing an adverse prognosis in already diagnosed patients. Even though CBD shows therapeutic effects and THC opposing effects, the data is minimal and low safety and efficacy warrants more research. The relation between cannabis and schizophrenia needs further investigation. We need more case-control studies and clinical trials with a larger population to get conclusive data.

## Introduction and background

In today’s stress-driven lives all around the world, mental health might be the most crucial aspect of overall health. People find different activities to feel better mentally and emotionally, some of them are creative and others destructive. One of these activities for mental relaxation is substance abuse. We all know many misused substances, but cannabis is one of the most widely used ones [1]. Cannabis is more commonly known as marijuana, hash, pot, blaze, herb, hay, or weed [2]. Smoking of dried plants in the form of a cigarette (aka joint), pipes, water-filled pipes (aka bong), or using it in edible products like cookies and brownies are some of the most preferred ways to use cannabis. However, now people also use it in the form of concentrated oil or vapor, mainly tetrahydrocannabinol (THC) [3]. The number of people who believe that regular marijuana use can be harmful is decreasing [3]. In 2007, 7% of the population, aged 15-64 in the Americas reported using marijuana [4], which increased to 8.4 percent in 2017 [5]. The most increase was observed in the United States of America, from 9.9% in 2007 to 15.3% in 2017 [6]. The most increase in cannabis use was among high school students [6]. The young population who do not attend college are more vulnerable to use marijuana daily [5].

Schizophrenia is a chronic mental condition affecting 20 million people worldwide [7]. Studies using household-based survey samples, clinical diagnostic interviews, and medical records show that the prevalence of schizophrenia and related psychotic disorders in the US is estimated to range between 0.25% and 0.64% [8]. Estimation of the international prevalence of schizophrenia among non-institutionalized persons is 0.33% to 0.75% [8]. Diagnostic and Statistical Manual of Mental Disorders, 5th edition (DSM-5) defines schizophrenia as two or more of either delusions, hallucinations, disorganized speech, disorganized behaviors, or negative symptoms present for a significant amount of time during one month among which at least one of these symptoms should be delusions, hallucinations, or disorganized speech, and the signs of disturbance must persist for at least six months [9]. Schizophrenia has a group of “positive” and “negative” symptoms. Positive symptoms are delusions, hallucinations, disorganized speech/ behavior/ thinking, and negative symptoms are diminished expressions, avolition, alogia, anhedonia, and asociality [10]. The presence of delusions or hallucinations is also known as a psychotic disorder [9]. Psychosis describes symptoms, whereas schizophrenia is a chronic illness that includes severe psychotic symptoms [10]. According to DSM-5, schizophrenia and brief psychotic disorder are a part of the schizophrenia spectrum, a group of disorders [9].

It is a well-known fact that many of the abuse substances cause psychosis, which is a part of the schizophrenia spectrum; cannabis is one [9]. Moreover, if cannabis use leads to schizophrenia development or worsens schizophrenic symptoms, it is a subject of interest for many psychiatrists and researchers. Researchers are conducting studies about its pharmaceutical use as well as its relation with schizophrenia and psychosis. Marijuana is legal for medical use in 31 states, and the District of Columbia in the United States [11] and nine states have legalized it for recreational use [12]. That is why it is imperative to understand how cannabis can affect our mental health. The purpose of this study is to know to what extent cannabis use and schizophrenia are related. We want to know if marijuana can be a causative factor for psychosis or schizophrenia.

In this systematic review, we will see marijuana’s relations with schizophrenia. We will try to understand if cannabis is causing schizophrenia or treating some of the schizophrenic symptoms. We will review multiple articles on marijuana/ cannabis and schizophrenia/ psychosis and try to understand their correlation. We plan to push our knowledge a little further at the end of this review.

## Review

Methodology

We conducted our systematic review using Preferred Reporting Items for Systematic Reviews and Meta-Analyses (PRISMA) guidelines.

Database

We started our research on March 24, 2020, using online libraries as our database. We searched PubMed, PubMed Central, Google Scholar, and Cochrane Library for our data collection

Search Strategy

We included studies related to marijuana/cannabis use and schizophrenia/psychosis. Our keywords and medical subject heading (MeSH) search strategies included marijuana, mental health, cannabis, schizophrenia, psychosis. We included the results for each search in Table 1.

**Table 1:** Initial search results This table represents the search result numbers that we found for each keyword or MeSH (Medical Subject Heading) keyword.

Keyword/ MeSH keyword	Database	Search result number
Cannabis	PubMed Central	14388
	PubMed	1352
	Google Scholar	67500
	Cochrane	37
Marijuana	PubMed Central	22081
	PubMed	1672
	Google Scholar	48800
	Cochrane	67
Mental health	PubMed Central	219934
	PubMed	24285
	Google Scholar	1110000
	Cochrane	2175
Marijuana, mental health	PubMed Central	12140
	PubMed	305
	Google Scholar	21800
	Cochrane	44
Marijuana, Schizophrenia	PubMed Central	4579
	PubMed	124
	Google Scholar	14700
	Cochrane	1
Marijuana, psychosis	PubMed Central	4961
	PubMed	180
	Google Scholar	13200
	Cochrane	1
Marijuana, schizophrenia, psychosis	PubMed Central	2977
	PubMed	77
	Google Scholar	9490
	Cochrane	1
Marijuana, cannabis, Schizophrenia, psychosis	PubMed Central	2469
	PubMed	73
	Google Scholar	5540
	Cochrane	1

Inclusion Criteria

We selected peer-reviewed articles and studies from the last five years published in the English language. We included only human studies in the category of systematic reviews, traditional reviews, meta-analysis, randomized trials. All data collected is ethical and legal.

Exclusion Criteria

We excluded gray literature and animal studies. We also excluded studies done before the last five years.

Quality Assessment Tools

We used a measurement tool to assess systematic reviews (AMSTAR) questionnaire for systematic reviews and meta-analysis, Cochrane risk bias assessment tools for clinical trials, New Castle-Ottawa questionnaire for observational studies, and the scale for the assessment of narrative review articles (SANRA) scale for traditional reviews. We excluded studies that were of low quality.

Data Collection

Data collection was done individually from the final articles after quality assessment.

Result

**Figure 1:**
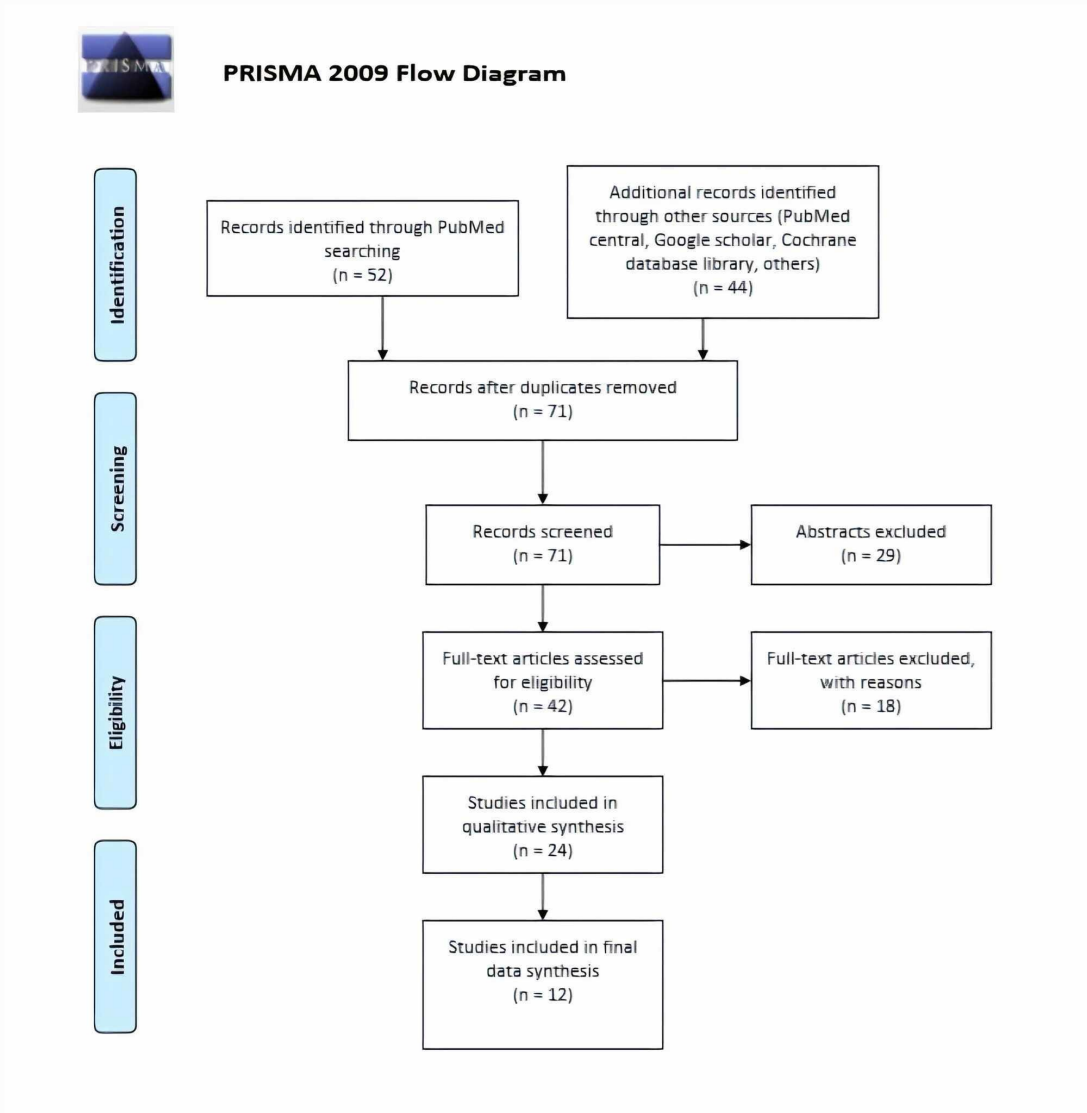
PRISMA flow diagram

After searching the database, we selected 96 studies after screening the titles and reviewed them. After screening the articles and removing 25 duplicates (13 from articles from PubMed, and 12 articles from other sources), we ended up with 71 articles (Figure 1). We found 29 abstracts without full articles and excluded them from our final list. After reviewing full articles, we excluded 18 articles because we found them not relevant to our study. We used various quality assessment tools for the remaining 24 articles. We excluded 12 low-quality articles and included 12 articles for data extraction. Table 2 shows the characteristics of the included 12 articles.

**Table 2:** Characteristics of the studies included in this system review GAD: generalized anxiety disorder, PTSD: post-traumatic stress disorder, ADHD: attention-deficit hyperactivity disorder, MDD: major depressive disorder, CBD: cannabidiol, THC: tetrahydrocannabinol

Author	Journal and publication year	Psychiatric disorder included	Conclusion	Type of research
Pearson et al. [10]	International Journal of Environmental Research and Public Health. 2019	Schizophrenia. cannabis-induced psychotic disorder	Causative, exacerbating	Traditional review
Singh et al. [13]	Indian J Med Res. 2017	Psychosis, bipolar disorder, cognitive functions	Causative, exacerbating	Traditional review
Gage et al. [14]	Psychol Med. 2017	Schizophrenia	Causative	Meta-analysis
Abush et al. [15]	Psychiatry Res Neuroimaging. 2018	Schizophrenia, psychosis, bipolar disorder	Causative, exacerbating	Observational study
Hahn [16]	Schizophr Bull. 2018	Schizophrenia, psychosis	Causative, exacerbating, CBD therapeutic	Traditional review
Hanna et al. [17]	Am J Drug Alcohol Abuse. 2017	Schizophrenia psychosis, depression, bipolar disorder, GAD	Causative, exacerbating, CBD therapeutic	Traditional review
Sarris et al. [18]	BMC Psychiatry. 2020	Anxiety, PTSD, depression, insomnia, schizophrenia, bipolar disorder, ADHD	THC causative, exacerbating, CBD therapeutic	Systematic review
Marconi et al. [19]	Schizophr Bull. 2016	Schizophrenia	Causative	Meta-analysis
Kelley et al. [20]	Schizophr Res. 2016	Schizophrenia, psychosis	Causative	Observational study
Hanna et al. [21]	Schizophr Bull. 2016	Schizophrenia, psychosis, bipolar disorder	Causative, exacerbating, CBD therapeutic	Observational study
Lowe et al. [22]	Eur Arch Psychiatry Clin Neurosci. 2019	Schizophrenia, GAD, bipolar disorder, MDD, PTSD	Exacerbating, CBD therapeutic	Traditional review
Hoch et al. [23]	Eur Arch Psychiatry Clin Neurosci. 2019	Schizophrenia, GAD, PTSD, Alzheimer’s, Tourette’s, anorexia nervosa, ADHD, cannabis dependence	CBD therapeutic	Systematic review

Study type

We included five traditional reviews, two systematic reviews, two meta-analyses, three observational studies in our final analysis.

Included Ailments

We had six records based solely on the relation between schizophrenia/ psychosis and marijuana/ cannabis. The other six records reviewed the link between marijuana/ cannabis and multiple psychiatric illnesses, including schizophrenia/ psychosis.

Outcome

Ten of the final records concluded marijuana as a causative factor for schizophrenia. Eight records found that marijuana, specifically tetrahydrocannabinol (THC) has an exacerbating effect on schizophrenia symptoms. Six records said that marijuana/cannabis derivative cannabidiol (CBD) could help in treating specific symptoms of schizophrenia.

Discussion

There are clear psychiatric comorbidity and cognitive decline in cannabis use disorder [13]. A person ever exposed to cannabis in his/her lifetime is at a higher risk of schizophrenia development [10]. There is a high frequency of psychotic disorders with cannabis users, and cannabis also alters the age of onset and course and presentation of the disease [13]. Cannabis-induced psychosis is a part of the schizophrenia spectrum that eventually convert to schizophrenia [10]. Patients with psychotic disorders have a higher tendency to use marijuana. A reverse causation hypothesis suggests that the diagnosis of schizophrenia predicts cannabis use initiation [14]. However, we lack information about their mechanism of action. Among psychotic/ schizophrenia patients, 16% currently use cannabis, and 27% have a history of use in their lifetime [15]. One-third to two-thirds of the psychotic population start using cannabis after the first psychotic break [16]. These numbers suggest that marijuana might be more pleasurable for schizophrenia spectrum patients, which is merely speculative and requires further investigation [14]. It can also be due to recall bias in many of the studies.

Nevertheless, the fact remains that schizophrenia and psychosis walk hand in hand alongside with cannabis use. Cannabis has many strains with different ratios of components. The ratio of THC and CBD is the most important psychotomimetic property of any cannabis strain [14]. When a healthy person uses cannabis, he experiences relaxation, euphoria, and a decrease in anxiety and boredom. However, they might also have some undesirable effects like paranoia, grandiosity, agitation, hallucination, cognitive impairment, disorganized thinking and behavior, and depersonalization [17]. People predisposed to the development of psychotic illness are more vulnerable to the psychotomimetic effects of cannabis, more specifically, THC [16,17].

Per se cannabis does not cause schizophrenia or psychosis. However, we have longitudinal data supporting the causal link between cannabis and psychosis [18]. The causal relationship is not explicit, but there is a minimum link connecting both [14,19]. The confounding factors to cannabis use and schizophrenia development can be gender, family history, genetic predisposition, and more [20]. Cannabis interacts with pre-existing genetic and environmental factors and leads to early schizophrenia [13,15,17,21]. A person who will inevitably develop a psychotic illness has generalized brain dysfunction, whereas a person who needs cannabis exposure with their genetic predisposition to develop psychosis has a more selective effect of psychosis and selective cognitive defect [21]. Frequent cannabis users have increased risk of developing psychosis/ schizophrenia and that too, at an earlier age [20]. The adolescent brain responds to cannabis differently than the adult brain [15]. The age when cannabis usage starts directly correlates with the age of onset of psychosis [20]. There is a positive association between cannabis dose and psychosis, increasing psychosis by four-fold in the heaviest users and two-fold in average users compared to non-users [19]. An increase in frequency and dose of cannabis use are most predictive of future psychosis/schizophrenia development [19,20].

Case-control studies show that earlier and higher doses of cannabis use lead to the more rapid development of psychotic symptoms [10,15]. Frequent use of cannabis, especially the start of use at a younger age, doubles the risk of schizophrenia development in the future [16]. Daily use of marijuana increases the risk of psychotic illness development with as much as five times higher risk in person using high potency THC [18]. We already have studies supporting genetic predisposition to schizophrenia. Brain-derived neurotrophic factor (BDNF), cannabinoid receptor 1 (CNR1), catechol-o-methyl transferase (COMT), protein kinase B also known as AKT1, and dopamine receptor D2 (DRD2) are some of the genes that are at risk of developing schizophrenia after early cannabis exposure; however, the transition rate is meager [18]. Cannabis changes the natural course of psychotic symptoms in the genotype Met/Met [10]. From this data, we can say that harm minimizing approach aiming at dose reduction or later onset of use can be relevant in the prevention and treatment of psychosis [19].

In laboratory studies, THC at high doses produces psychosis-like symptoms and shows a transient increase in positive symptoms and cognitive impairment. In contrast, CBD attenuates adverse effects caused by THC [17, 18]. Among chronic users, comparison of cognitive effect between weaker and stronger preparation users showed a decline among stronger preparation users in the form of poor concentration and attention, memory impairment [13]. Younger and more frequent users are at higher risk of developing cognitive decline [13]. Adolescent cannabis use impacts cognition in the future [21]. Imaging shows more significant brain matter loss in first-episode schizophrenia patients with a history of cannabis use [21]. Cannabis use after the first psychotic break is a significant predictor for poor prognosis because of low adherence and inadequate response to antipsychotics seen in cannabis users [16]. Heavy and chronic cannabis use after schizophrenia spectrum diagnosis can lead to more and earlier relapses with worsening of symptoms and more extended hospitalization even in patients stable on antipsychotics [16,17,22]. Effect of cannabis use on cognitive outcomes are controversial as acute effects of THC mimics positive, negative and cognitive symptoms of schizophrenia along with neurophysiological phenomena of psychosis in non-psychotic person and worsens psychotic symptoms and cognitive function in schizophrenic person but CBD use shows a better cognitive function in the schizophrenic person [16]. THC increases psychosis symptoms in a dose-dependent fashion, primarily IV THC [22].

Even after diagnosis, chronic cannabis use in schizophrenic patients has a detrimental effect on brain morphology [16]. Neuroimaging review by Abush H et al. can be a significant discovery in understanding cannabis use effect on adolescent brain and schizophrenia development. Their review shows a whole-brain and regional gray matter density (GMD) decrease in schizophrenic and psychotic patients, both with adolescent cannabis use history and without adolescent cannabis use history. However, GMD is less reduced in psychosis with a history of early cannabis use history [15]. Gray matter density and cognition in schizophrenia with adolescent cannabis use is similar to healthy control, which suggests that they had low vulnerability for psychosis, and perhaps had they not used cannabis, they would not have developed psychosis [15]. They may represent a clinical subgroup with novel illness mechanism which requires novel therapies [15]. Early cannabis use is also associated with increased cortical thickness and gray/ white matter border contrast [15,21]. They also have decreased local gyrification index in the prefrontal cortex [15]. Chronic low dose exposure to THC modified dendrites in the shell of the nucleus accumbens and medial prefrontal cortex, resulting in increased dendritic length and increased numbers of branches [15]. These data help us formulate two theories to explain the GMD difference in patients with cannabis use history and no cannabis use. One, cannabis abuse can precipitate psychosis in person with less prominent cognitive and neuro-anatomical risk factors for illness. Two, cannabis has a sparing effect on brain structure [15]. However, to have definite answers, we need more case-control studies.

On the other hand, we have data showing the therapeutic effects of cannabis. Many trials are going on right now on CBD and its use in psychiatric illnesses. Schizophrenia spectrum patients with a history of cannabis use in their adolescence show better cognitive performance on BACS composite score compared to patients with no adolescent cannabis use history [15,21]. This difference can be due to CBD in cannabis. Compared to THC, CBD has subtle subjective effects and no euphorigenic properties, and it also reduces the efficacy of THC [16]. In one trial, 600-800 mg CBD for four weeks in schizophrenic patients alleviated psychotic symptoms similar to antipsychotic drug amisulpride but with fewer side effects [16]. In another trial, 1000 mg CBD for six weeks in adjunction to antipsychotic medication in stable schizophrenia spectrum diagnosed people showed a reduction in positive symptoms [16,18,22,23]. A study has shown that 1500 mg CBD daily for 26 days is beneficial in treatment-resistant schizophrenia [18]. Three RCTs showed improvement with CBD in both cognition and psychotic symptoms in patients with schizophrenia spectrum diagnosis [23]. These CBD properties can be useful for the tailored intervention of psychosis and schizophrenia with co-morbid cannabis misuse [16]. It may even reduce cannabis use itself by lowering the psychotomimetic effect of THC [16]. Even though CBD shows improvement in positive affect in psychotic individuals, it also increases hallucinations in some cases and worsens negative symptoms [17]. Even after CBD’s therapeutic potential, low efficacy, and safety of cannabis-based medications warrant more extensive trials for more data [23].

Even after all the available data, our systematic review has limitations. We only included data published in the English language from the last five years. We excluded articles other than systematic reviews, traditional reviews, meta-analysis, and randomized trials. We only included human trials and did not include gray literature and non-peer-reviewed articles. There can be recall bias in some of the studies included. We need further research on the neurobiological aspects of cannabis. More clinical trials and case-control studies with a larger population are required for more detailed data before we can get a final result. We should study all the possible health effects in proper depth. With the knowledge that we have right now, use in at-risk adolescent populations with developing brains should be discouraged. Furthermore, even in a population that uses cannabis, proper education about its effect is needed.

## Conclusions

Cannabis and schizophrenia/psychosis have a close relationship. We have evidence suggesting that cannabis use, primarily THC in cannabis, in genetically predisposed or at-risk populations, leads to earlier diagnosis of psychosis/schizophrenia. This tells us that THC in cannabis has a small causative effect on schizophrenia. THC in cannabis also makes schizophrenia and psychosis symptoms worse and causes more relapses and hospitalizations. Neuroimaging studies show the detrimental effect of cannabis on brain morphology, especially adolescent brains. Recent trials in therapeutic CBD use are showing its alleviating effect on positive symptoms of schizophrenia and its opposing effect on THC, which warrants further research. Although CBD shows therapeutic potential, there is still more harm from cannabis than benefits, and adolescent cannabis usage should be discouraged at all costs. We still need more extensive studies for more detailed data about cannabis and its effects.
